# A method of producing genetically manipulated mouse mammary gland

**DOI:** 10.1186/s13058-018-1086-8

**Published:** 2019-01-05

**Authors:** Hiroaki Tagaya, Kosuke Ishikawa, Yoshito Hosokawa, Shun Kobayashi, Yukino Ueoka, Mayuna Shimada, Yasuko Ohashi, Hirofumi Mikami, Mizuki Yamamoto, Tatsuya Ihara, Kentaro Kumazawa, Kosuke Sugihara, Naoki Goshima, Shinya Watanabe, Kentaro Semba

**Affiliations:** 1 Department of Life Science and Medical Bioscience, School of Advanced Science and Engineering, Waseda University, 2-2 Wakamatsu-cho, Shinjuku-ku, Tokyo, 162-8480 Japan; 2 Computational Bio Big-Data Open Innovation Laboratory (CBBD-OIL), National Institute of Advanced Industrial Science and Technology (AIST), 3-4-1 Okubo, Shinjuku-ku, Tokyo, 169-8555 Japan; 3 Japan Biological Informatics Consortium (JBiC), 2-45 Aomi, Koto-ku, Tokyo, 135-8073 Japan; 4 Division of Cellular and Molecular Biology, The Institute of Medical Science, The University of Tokyo, Tokyo, Japan; 5 National Institute of Advanced Industrial Science and Technology (AIST), Koto-ku, Tokyo, 135-0064 Japan; 6 Translational Research Center, Fukushima Medical University, Hikarigaoka, Fukushima, 960-1295 Japan

**Keywords:** *PiggyBac*, Transposon, Transgenesis, Electroporation, MaSC, Doxycycline, Tet-On system

## Abstract

**Background:**

To obtain a deep understanding of the mechanism by which breast cancer develops, the genes involved in tumorigenesis should be analyzed in vivo. Mouse mammary gland can regenerate completely from a mammary stem cell (MaSC), which enables us to analyze the effect of gene expression and repression on tumorigenesis in mammary gland regenerated from genetically manipulated MaSCs. Although lentiviral and retroviral systems have usually been applied for gene transduction into MaSCs, they are associated with difficulty in introducing long, repeated, or transcriptional termination sequences. There is thus a need for an easier and quicker gene delivery system.

**Methods:**

We devised a new system for gene delivery into MaSCs using the *piggyBac* transposon vectors and electroporation. Compared with viral systems, this system enables easier and quicker transfection of even long, repeated, or transcriptional termination DNA sequences. We designed gene expression vectors of the transposon system, equipped with a luciferase (Luc) expression cassette for monitoring gene transduction into regenerative mammary gland in mice by in-vivo imaging. A doxycycline (Dox)-inducible system was also integrated for expressing the target gene after mammary regeneration to mimic the actual mechanism of tumorigenesis.

**Results:**

With this new gene delivery system, genetically manipulated mammary glands were successfully reconstituted even though the vector size was > 200 kb and even in the presence of DNA elements such as promoters and transcription termination sequences, which are major obstacles to viral vector packaging. They differentiated correctly into both basal and luminal cells, and showed normal morphological change and milk production after pregnancy, as well as self-renewal capacity. Using the Tet-On system, gene expression can be controlled by the addition of Dox after mammary reconstitution. In a case study using polyoma-virus middle T antigen (PyMT), oncogene-induced tumorigenesis was achieved. The histological appearance of the tumor was highly similar to that of the mouse mammary tumor virus-PyMT transgenic mouse model.

**Conclusions:**

With this system, gene transduction in the mammary gland can be easily and quickly achieved, and gene expression can be controlled by Dox administration. This system for genetic manipulation could be useful for analyzing genes involved in breast cancer.

**Electronic supplementary material:**

The online version of this article (10.1186/s13058-018-1086-8) contains supplementary material, which is available to authorized users.

## Background

Most breast cancers originate from mammary epithelial cells (MECs) and progress through multiple steps. When a tumorigenic event such as gene amplification occurs [1], MECs acquire the potential to proliferate in the mammary duct and develop into ductal carcinoma in situ (DCIS) [2], which progresses to malignant carcinoma such as invasive breast carcinoma and then metastasizes into other organs through mesenchymal tissue. For in-vivo analysis of the genes involved in these processes, some genetically manipulated mouse models have been established, such as *TP53*^−/−^, mouse mammary tumor virus (MMTV)-*neu*, and MMTV-polyoma-virus middle T antigen (PyMT) mice. These models have boosted our understanding of the mechanisms by which mammary tumors develop and metastasize in vivo [3–5]. We have identified several oncogene candidates from amplicons of breast cancer cell lines and showed that some of the genes exhibited tumorigenic activity when gene-introduced cells were inoculated subcutaneously into nude mice [6–9]. Transgenic or knockout mouse models are useful for understanding the tumorigenic activity of those genes, but have not been practical for analyzing multiple candidate genes. Therefore, we have to establish a novel alternative method for preparing genetically engineered mice to improve the efficiency for analysis of multiple genes.

The mammary gland is a unique organ in that most mammary development occurs postnatally [10]. A small fraction of mammary stem cells (MaSCs) and progenitor cells in the basal layers of the mammary gland have regenerative capacity and maintain organ homeostasis during estrous cycles [11–17]. It has been demonstrated that the transplantation of a cell fraction with the surface markers CD49f^high^CD24^+^ or CD29^high^CD24^+^ into mouse fat pads from which the endogenous epithelium has been removed (cleared fat pads) can regenerate fully functional mammary gland [12, 13].

Harnessing this regenerative capacity of MaSCs, gene-introduced mouse mammary gland can be produced from cells enriched in MaSCs by infection with lentiviral or retroviral vectors, which is a valuable alternative approach to producing transgenic animals [18, 19]. However, the efficiency of viral packaging has been lower when introduced genetic elements have a longer or more complicated sequence, including promoter and transcription termination sequences. In addition, to mimic the actual mechanism of tumorigenesis, the target gene is required to be inducibly expressed after mammary reconstitution.

Here, we describe a convenient method for the genetic manipulation of mouse mammary gland using a transposon vector system and electroporation. Using this method, mCherry-expressing mammary glands were first generated. The tissues were analyzed by immunostaining to examine the transgene distribution in both luminal and basal cells. Milk production during pregnancy was also examined to verify normal differentiation and mammary gland function. In addition, a vector with long DNA (> 200 kb) derived from a bacterial artificial chromosome (BAC) clone was introduced to test its loading capacity. Moreover, using doxycycline (Dox)-inducible expression vector, we assessed the Dox-dependent expression of enhanced green fluorescent protein (EGFP) after mammary gland reconstitution. To examine whether oncogene- induced tumorigenesis is achieved with this method, *PyMT* was chosen for a case study and histological analyses were conducted to compare its mammary tissue with that of the MMTV-PyMT transgenic mouse model.

## Methods

### Mice

For a transplantation assay, *rag2*^*−/−*^ (kindly provided by Dr. Takaki) immunocompromised albino mouse lines backcrossed with FVB or C57BL/6 J were used as recipient mice. FVB or C57BL/6 J mice were used as donors.

### Vector construction and preparation

All vectors were constructed using either a ligase reaction kit (Nippon Gene, Tokyo, Japan, or Takara, Kyoto, Japan) or the In-Fusion reaction kit (Takara). In the case of long transposon donor vector loading BAC (Fig. 5a), a template vector was first constructed by joining six fragments using an In-Fusion reaction after providing each fragment with polymerase chain reaction or annealing 2-oligo DNAs. The contents of this template vector are depicted at the bottom of Fig. 5a. Here, 70-bp and 134-bp arms homologous to the BAC vector (pBACe3.6) were placed in its flanking regions. The N*ot*I/B*st*XI (FastDigest from Thermo Scientific)-digested fragment of this template vector was introduced into an *E. coli* BAC clone B6Ng01-263 N07 (RIKEN BioResource Center (BRC)) by electroporation, and a homologous recombination reaction was conducted by the RED/ET system (Gene Bridges, Heidelberg, Germany) to obtain the long transposon donor vector (Fig. 5a).

The long transposon donor vector was then purified using the NucleoBond Xtra BAC kit (Macherey-Nagel, Takara). Other vectors were purified with CsCl-gradient ultracentrifuge sedimentation after purification with an alkaline lysis solution method.

### Cell culture

Mouse embryo-derived fibroblasts, C3H10T1/2 (RCB0247; RIKEN BRC), were cultured in Dulbecco’s modified Eagle’s medium (DMEM; Wako Pure Chemical Industries, Ltd., Tokyo, Japan) containing 5% fetal bovine serum (FBS), 100 μg/mL streptomycin sulfate (Meiji Seika Pharma, Tokyo, Japan), and 100 U/mL penicillin G potassium (Meiji Seika Pharma) at 37 °C and 5% CO_2_. For co-culture with MEC, C3H10T1/2 cells were treated with 4 μg/mL Mitomycin C (Wako) for 3 h and incubated overnight at 5% CO_2_ and 37 °C for 1–2 days.

NMuMG cells were cultured in DMEM supplemented with 10% FBS, 100 μg/mL streptomycin sulfate, 100 U/mL penicillin G potassium, 10 μg/mL insulin, and 0.45% glucose.

### Mammary cell preparation

The thoracic, abdominal, and inguinal mammary glands were dissected from 8- to 10-week-old female donor mice. After washing in phosphate-buffered saline (PBS), the tissues were chopped with a razor. Then, the tissues were digested for 1–2 h at 37 °C under shaking in DMEM/F12 medium (Invitrogen, Carlsbad, CA, USA) containing 5% FBS, 2 mg/mL collagenase IV (Sigma, Poole, UK), and 0.1 mg/ml hyaluronidase (Sigma). After removing the red blood cells in DMEM/F12 by repeated low-gravity centrifugation followed by suspension in ACK buffer (0.15 M NH_4_Cl, 10 mM KHCO_3_, 0.1 mM EDTA, pH 7.3), a dissociated cell suspension was yielded by pipetting for 8–10 min in cell dissociation buffer (PBS, 0.05% trypsin, 0.5 mM EDTA, 0.5% DNaseI), and then for 2 min in 5 mg/mL dispase (StemCell Technologies, Vancouver, Canada)–0.5% DNaseI, followed by filtration through a 42-μm mesh.

### Cell sorting

A dissociated single-cell suspension was labeled with biotinylated CD45, Ter119, CD31, and BP-1 antibodies contained in EasySep Mouse Epithelial Cell Enrichment Cocktail (StemCell Technologies) for 30 min on ice, and after washing, incubated with anti-CD49f-PE (eBioscience, San Diego, CA, USA), anti-CD24-perCP-cy5.5 (eBioscience), streptavidin-ECD (Beckman-Coulter, Brea, CA, USA), and 7-amino-actinomycin D (7-AAD; Calbiochem, San Diego, CA, USA) for 30 min on ice. Cells were suspended in 2% FBS–Hank’s balanced salt solution including DNaseI before sorting. Cell sorting was carried out using fluorescence-activated cell sorting (FACS) SH800 (Sony, Tokyo, Japan). CD45^−^ Ter119^−^ CD31^−^ BP-1^−^ (Lin(−)) 7-AAD^−^ CD49f^high^ CD24^+^ cells were collected to represent the MaSC-enriched fraction.

### Transfection

A total of 1 × 10^6^ cells of the sorted MaSC-enriched fraction from about eight mice were suspended and washed in Opti-MEM (Life Technologies, Carlsbad, CA, USA) twice. Then, 10 μg of donor and helper vector DNA were mixed at a ratio of 3:1 with the MECs in Opti-MEM, and subjected to electroporation using NEPA21 (NEPAGENE, Chiba, Tokyo) with a 2-mm gap electrode cuvette (NEPAGENE, EC-002S). The settings for this electroporation are shown in Additional file 1 (Table S1). After electroporation, the cell suspension was immediately suspended in culture medium.

For transfection into NMuMG, dissociated cells were plated on six-well plates at 20–30% confluence a day before transfection. Donor and helper vectors were introduced at an OD_260_ ratio of 3:1 by pouring the mixture of 4 μg of DNA and 8–12 μg of polyethylenimine (Polysciences, Warrington, PA) into 200 μL of Opti-MEM.

### Culture system for maintaining stemness of MaSCs

MECs were co-cultured with mitomycin C-treated C3H10T1/2 in DMEM/F12 supplemented with 10% FBS, 10 ng/mL human epidermal growth factor (hEGF; BD Biosciences, Tokyo, Japan), 5 μg/mL insulin (Wako), 0.5 μg/mL hydrocortisone (Sigma), 5 μM forskolin (Wako), 1.8 × 10^−4^ M adenine (Sigma), 100 μg/mL streptomycin (Meiji Seika Pharma), 100 U/mL penicillin G (Meiji Seika Pharma), 50 μg/mL gentamycin (Nakalai Tesque, Tokyo, Japan), 10 μM Rho-associated coiled-coil-forming kinase inhibitor (ROCKi; Y-27632; LC Laboratories, New Boston, MA, USA) [20, 21], and 10% Matrigel (growth factor reduced; BD Biosciences) at 5% CO_2_ and 37 °C for 7 days. The numbers of MECs and C3H10T1/2 were 5000 and 6.25 × 10^4^, respectively, in 250 μL of culture medium in a 48-well plate. When wells of different sizes were used, these numbers were changed proportionately relative to the area of the well.

### Transplantation assay

The transfected MECs under culture were treated with dispase for 1–2 h at 37 °C and then approximately 3% of all transfected cells were suspended in 4–10 μL of DMEM/F12 including 10% Matrigel per transplantation site and transplanted into the cleared fat pads of the inguinal mammary glands of *rag2*^−/−^ mice from which the endogenous epithelium had been removed using a 50-μL syringe equipped with a 30-G needle (ITO, Shizuoka, Japan). Mammary repopulation was analyzed by detecting bioluminescence using an in-vivo imaging system (IVIS Lumina XR, PerkinElmer, Waltham, MA, USA) and mCherry fluorescence using a stereomicroscope (Leica, Wetzlar, Germany).

### Carmine alum staining

The dissected mammary glands were spread on a glass slide and fixed using Carnoy’s fixative (60% ethanol, 30% chloroform, and 10% glacial acetic acid) overnight at room temperature. Fixed tissues were washed in 70% ethanol, gradually rehydrated to distilled H_2_O, and then incubated in carmine alum solution (0.2 wt% of carmine (Sigma) and 0.5 wt% of aluminum potassium sulfate (Wako) in distilled H_2_O) for 2 h to overnight at room temperature. After gradual dehydration from 70% ethanol to 100% ethanol, fat pads were cleared overnight in xylene and mounted in MGK-S (Matsunami, Osaka, Japan).

### Hematoxylin and eosin staining

After washing in PBS, the dissected mammary glands and tumors were fixed in 4% paraformaldehyde–PBS overnight and for 2 days, respectively. After washing in PBS, samples were dehydrated gradually from 70% ethanol to 100% ethanol and then from 50% xylene in ethanol to 100% xylene, following paraffin replacement using a Leica ASP300 fully automatic closed tissue processor and paraffin-embedding using a Leica EG1160. The paraffin-embedded tissues were then cut into 5-μm thick sections using a Leica SM200R sliding microtome. The sections were gradually deparaffinized in xylene and then with ethanol, gradually decreasing from 100% to 50% ethanol, and washed with distilled H_2_O, and then stained in hematoxylin solution (0.25% hematoxylin (Nacalai Tesque), 0.05% sodium iodate (Nacalai Tesque), 12.5% potassium alum (Wako), and 0.25% citric acid (Wako)) for 10 min. After washing in distilled H_2_O, sections were blued in 0.1% saturated lithium carbonate at 37 °C for 5 min and then washed in distilled H_2_O and stained in eosin solution (1% eosin (Wako) and 0.02% glacial acetic acid) for 10 min. After washing in 90% and 100% ethanol, sections were soaked in xylene for 5 min and then mounted in MGK-S (Matsunami).

### Immunohistochemistry

The inguinal mammary glands were dissected, cut into ~ 1–5-mm^3^ fragments, and pre-fixed for 10–15 min in 4% paraformaldehyde–PBS on ice. Tissues were washed in cold PBS and incubated for about 1 h in 10% sucrose–PBS at 4 °C, for about 1 h in 20% sucrose–PBS at 4 °C, and overnight in 40% sucrose–PBS at 4 °C. Tissues were frozen in cryoembedding medium [22] in liquid nitrogen. The frozen blocks were then cut into sections of 5 or 10 μm thickness using a Leica CM1850 cryostat. Sections were dried for 1–10 min at room temperature, placed for more than 10 min in PBS, and post-fixed for 10–15 min in 3% paraformaldehyde at room temperature. After washing for more than 10 min in PBS or 0.1% Tween-PBS, sections were incubated in blocking buffer (0.1% Triton/10% goat serum in PBS) for 1 h at room temperature or overnight at 4 °C. Staining with primary antibodies was performed overnight at 4 °C or for 1 h at room temperature. Then, sections were washed three times in PBS or 0.1% Tween-PBS for 10 min, and staining with secondary antibody solutions containing 4′,6-diamidino-2-phenylindole (DAPI) was performed for 1 h at room temperature. In the case of milk fat globule (MFG) staining, sections were incubated in BODIPY 493/503 (3 μg/mL; Molecular Probes) containing DAPI in PBS for 10 min at room temperature. Then, sections were washed twice in PBS for 10 min. Finally, slides were mounted in MOWIOL DABCO.

The following primary antibodies were used: anti-KRT14 (mouse, 1:1000; Novocastra, Newcastle, UK), anti-KRT8 (rat, 1:250; Developmental Studies Hybridoma Bank, University of Iowa), anti-mCherry (rabbit, 1:250; Abcam, Cambridge, UK), and anti-PyMT (rat, 1:500; Santa Cruz, CA, USA). The following secondary antibodies were also used: anti-mouse, anti-rabbit, and anti-rat conjugated to AlexaFluor 488 (1:1000; Molecular Probes, Eugene, OR, USA) and to AlexaFluor 568 (1:1000; Molecular Probes). Staining of nuclei was performed with DAPI (1:4000; DOJINDO, Tokyo, Japan). Adjustment for the variations in the brightness and/or contrast of the entire area of images was performed using Photoshop (Adobe, San Jose, CA, USA).

### Immunoblotting

Cells were collected in RIPA buffer (10 mM Tris-HCl (pH 8.0), 1% (w/v) NP40, 0.1% (w/v) sodium deoxycholate (Wako), 0.1% (*w/v*) SDS (Wako), 0.15 M NaCl (Wako), 1 mM EDTA, 10 mM NaF (Wako), 1.5 mM Na_3_VO_4_ (Wako), and cOmplete™ Protease Inhibitor Cocktail (Roche)). Protein concentrations were titered using the BCA protein assay kit (Thermo Fisher Scientific). Collected protein lysate was mixed with SDS-PAGE loading buffer (0.15 M Tris-HCl, 6% (w/v) SDS, 0.003% (w/v) bromophenol blue (Wako), 30% (w/v) glycerol (Wako), and 15% (w/v) β-mercaptoethanol (Wako)) and then boiled at 95 °C for 5 min, followed by SDS-PAGE and immunoblotting. Antibodies used for immunoblotting were as follows: anti-milk (rabbit, 1:1000; Nordic-MUbio, Susteren, the Netherlands), anti-PyMT (rat, 1:500; Santa Cruz), anti-mCherry (rabbit, 1:500; Abcam), anti-histone H3 (rabbit, 1:2500; Cell Signaling Technology, MA, USA), and anti-α-tubulin (mouse, 1:5000; Calbiochem).

## Results

### *piggyBac* transposon vector system and electroporation enabled us to establish gene-introduced mammary glands

**Fig. 1:**
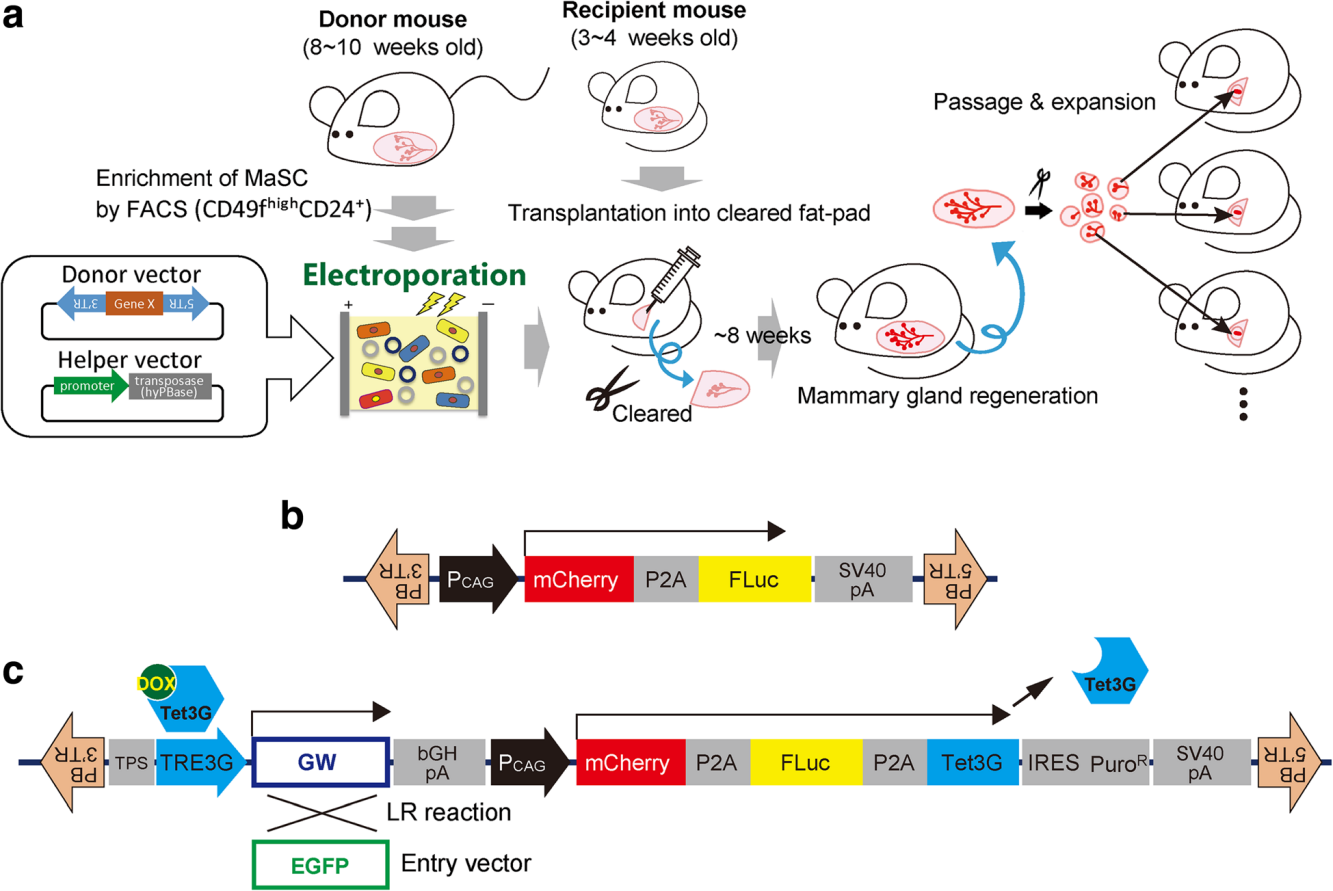
The mammary reconstitution and transposon vector system to analyze gene function in vivo. **a** Schematic view of vector introduction and expansion of mammary gland. **b** Vector construct of *piggyBac* transposon vector expressing mCherry and firefly luciferase (Fluc). **c** Vector construct of *piggyBac* transposon vector with TRE3G–Gateway elements. All vectors that we produced and predicted full sequences are available upon request. Dox doxycycline, EGFP enhanced green fluorescent protein, FACS fluorescence-activated cell sorting, MaSC mammary stem cell

For the efficient establishment of gene-introduced mammary gland, we developed a convenient method combining the *piggyBac* transposon vector system and electroporation (Fig. 1a). The transposon vector system has recently been broadly applied as a tool for transgenesis [23], including in breast cancer research [24]. Unlike the viral vectors, the transposon vector does not need the packaging cell for vector preparation. DNA elements between the inverted terminal repeat (ITR) sequences of transposon can be integrated into the host genome via a cut-and-paste mechanism by the expression of transposase. Fairly long DNA, even 150-kb BAC, can be transferred into the host genome [25–27]. With this approach, it is particularly useful to integrate the use of the *piggyBac* transposon system, which allows efficient gene delivery in mammalian cells [28–30]. These features allow us to introduce a DNA sequence with a termination or promoter sequence that could be an obstacle for packaging viral vectors efficiently into the host genome. Using electroporation, we can introduce the vectors into cells efficiently, easily, and rapidly. As an alternative to electroporation, lipofectamine is a possible option for quick and easy transfection although we have not compared these two approaches.

Here, we use the term “helper vector” to refer to a vector expressing transposase (we use a hyperactive mutant, hyPBase [29]), and use the term “donor vector” to refer to a vector having a designed DNA element between ITRs. First, two donor vectors were constructed: one expresses mCherry and firefly luciferase (Fluc) constitutively (Fig. 1b), and the other additionally expresses a gene of interest after normal mammary regeneration to mimic the actual mechanism of tumorigenesis by the Tet-On 3G system (Fig. 1c). Fluc and mCherry marker genes were placed downstream of the cytomegalovirus early enhancer/chicken β-actin (CAG) promoter that is useful for monitoring the regeneration of gene-introduced mammary glands. Under the same promoter, Tet3G gene of the Tet-On 3G system was also placed via a sequence encoding P2A peptide. TRE3G-Gateway elements, into which a gene of interest can be transferred from an ENTRY vector by a recombinase, called LR clonase, were placed on the same vector to express the gene in an inducible manner (Fig. 1c). These elements could simplify the construction of expression vectors for the genes of interest.

**Fig. 2:**
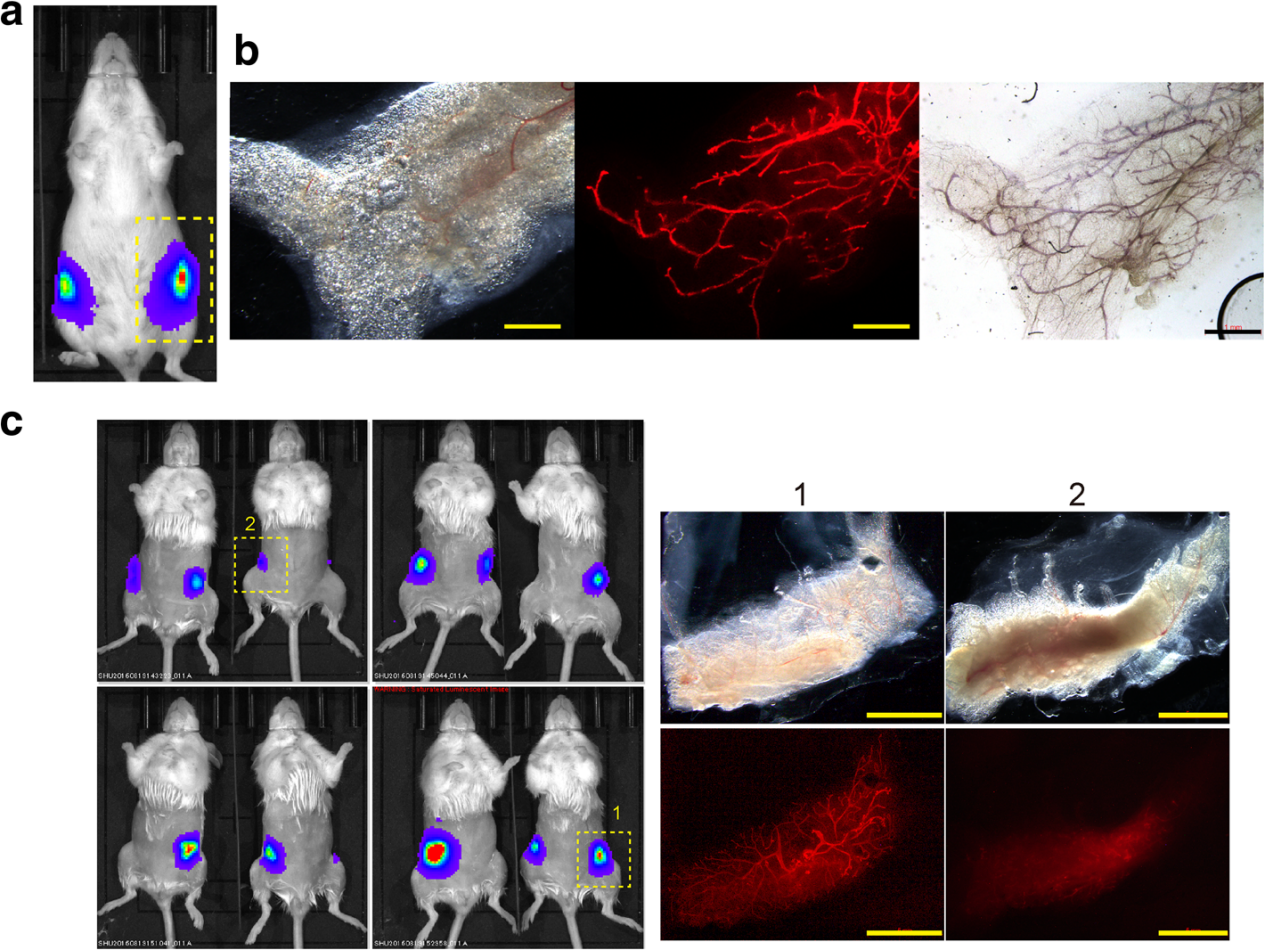
Establishment of gene-introduced mammary glands. **a** A representative image showing luciferase activity of primary outgrowths at day 56 after mammary epithelial cell injection into cleared fat pad. **b** Microscopic view of resected fat pads from **a**. Red fluorescence (middle panel) shows mCherry marker expression. Right panel shows carmine staining to show whole mammary glands in this fat pad. Scale bar = 1 mm. **c** IVIS images of secondary outgrowth at day 75 after transplantation of mCherry-expressing fragments of one primarily reconstituted mammary gland. Right panels are microscopic images of the dissected mammary gland from the site indicated by the yellow dotted boxes in the left images. Lower panels show mCherry marker expression. Scale bars = 5 mm

To produce gene-introduced mammary glands, the MaSC-enriched fraction (CD49f^high^ CD24^+^) was collected by FACS from mammary glands of donor female mice aged from 8 to 10 weeks (Fig. 1a, Additional file 1: Figure S1). Then, the donor and helper vectors of the *piggyBac* transposon system were co-introduced into the cells using electroporation, followed by in-vitro culture. There are various approaches to maintain the stemness of MaSCs in vitro [14, 31–34], and we chose one that uses Matrigel, fibroblasts, and ROCKi (see Methods); however, it is unknown whether this is the optimal approach. To test whether the Tet-On inducible system works properly, MECs into which the TRE3G-EGFP expression vector (Fig. 1c) had been introduced by electroporation were treated Dox during the culture. mCherry-labeled MECs correctly inducibly expressed EGFP in a Dox-dependent manner (Additional file 1: Figure S2). The gene-introduced MECs were collected from the culture and transplanted into the cleared fat pads of the inguinal mammary glands of *rag2*^−/−^ mice. After 6–12 weeks, luciferase marker expression was detected using an in-vivo imaging system (IVIS) (Fig. 2a, Table 1).

**Table 1:** Frequency of regeneration of gene-introduced mammary gland

Mammary regeneration	First generation	Second generation
First experiment	7/18	11/16
Second experiment	3/18	12/14

Mammary regeneration was judged from in-vivo imaging system analysis on the basis that > 1 × 10^6^ photons/s of total flux is positive and partially from microscopic analysis on the basis of mCherry fluorescence. A fragment of primary mammary product (first generation) was dissected on day 85 (first experiment) or 105 (second experiment) after transplantation, cut into pieces, and then transplanted into the fat pad of newly prepared mice, and the secondary outgrowths (second generation) were allowed to expand for 75 (first experiment) and 28 days (second experiment)

In the analysis of the resected fat pads, mammary outgrowth with mCherry expression was observed (Fig. 2b). The level of luciferase expression roughly correlated with the size of the mCherry-expressing mammary glands.

**Fig. 3:**
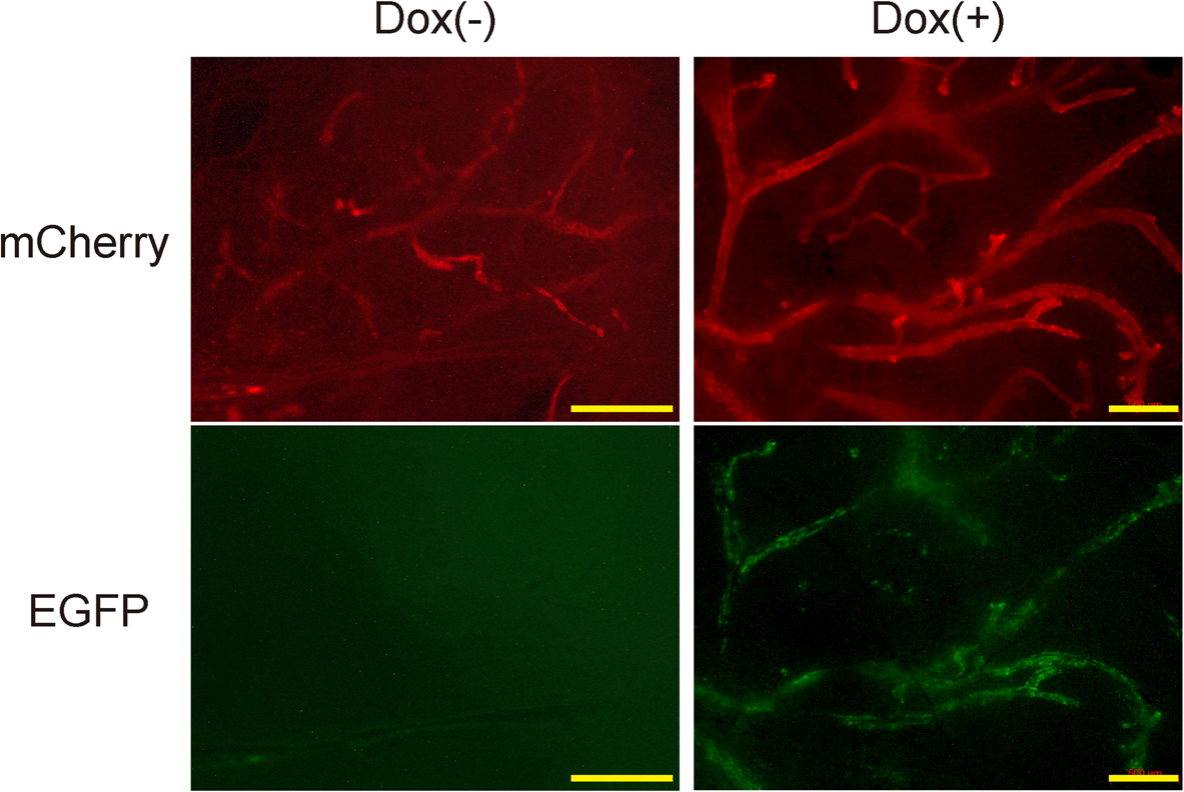
Dox-dependent expression of the TRE3G–EGFP-introduced mammary gland. Mice from Fig. 2c were exposed to doxycycline (Dox) (right) via drinking water for 55 days and compared to mice not administered Dox (left). Scale bars = 500 μm. EGFP enhanced green fluorescent protein

To evaluate the self-renewal capacity of the gene-introduced mammary glands, one of the mCherry-expressing portions (approximately 2 cm^2^) was cut into small pieces (3–5 mm^2^), which were transplanted into newly prepared *rag2*^−/−^ immunocompromised mice (Fig. 1a). Outgrowth of the secondary mammary glands was observed (Fig. 2c, Table 1), suggesting that the gene-introduced mammary gland had the capacity for self-renewal. We then exposed the mice to Dox via drinking water and compared their EGFP expression to mice without Dox administration, into both of which the same branch of transgenic mammary gland had been transplanted. We observed that EGFP was expressed only in Dox-administered mice (Fig. 3), suggesting that the gene expression can be specifically controlled in a Dox-dependent manner.

**Fig. 4:**
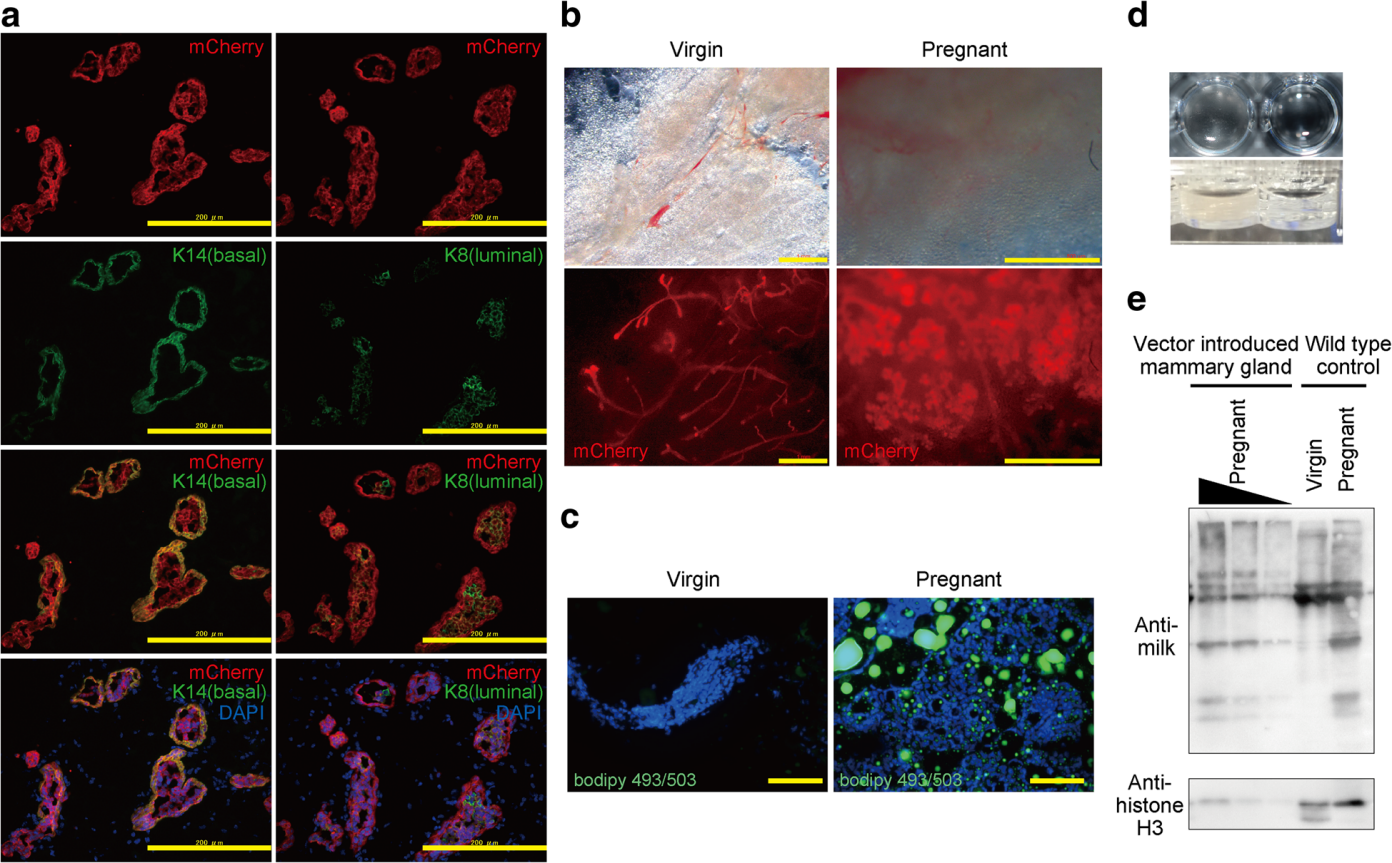
Analyses of the differentiation and function of gene-introduced mammary glands. **a** Lineage distribution of gene-introduced MECs. Co-immunostaining of mCherry (red, upper panels) in combination with K14 (basal marker, green, left panels) or K8 (luminal marker, green, right panels) in pregnant mice. Scale bars = 200 μm. **b** Gene-introduced outgrowths in virgin (left panels) and pregnant mice (right panels). Red fluorescence (lower panels) represents mCherry expression. Scale bars = 1 mm. **c** Analysis of milk production by staining of lipid droplets (BODIPY 493/503) of gene-introduced mammary glands during pregnancy (right panel). Left panel shows control staining of virgin mammary glands. Scale bars = 200 μm. **d** Turbid secretory components were observed upon the immersion of dissected regenerative mammary gland of pregnant mice in PBS (left). Right shows PBS control. **e** Immunoblot analysis of indicated lysates derived from virgin or pregnant mammary glands. Histone H3 represents internal control. DAPI 4′,6-diamidino-2-phenylindole

To confirm that the transgene was properly distributed to both mammary epithelial lineages, namely luminal and basal cells, we examined the tissue by histochemical analyses. We detected mCherry expression in both basal (positive for K14) and luminal (positive for K8) cell layers (Fig. 4a). In addition, pregnancy was found to result in morphological changes to the highly branched ductal and alveolar network (Fig. 4b) and the production of milk, which was assumed to occur from the detection of MFGs (Fig. 4c), opacity of secretory components (Fig. 4d), and the same pattern of milk protein as wild-type pregnant mammary gland (Fig. 4e).

These observations confirmed that the gene-introduced mammary glands exhibited normal differentiation potential and were fully functional.

### The *piggyBac* transposon vector system had a high capacity for loading long DNA elements (> 200 kb)

**Fig. 5:**
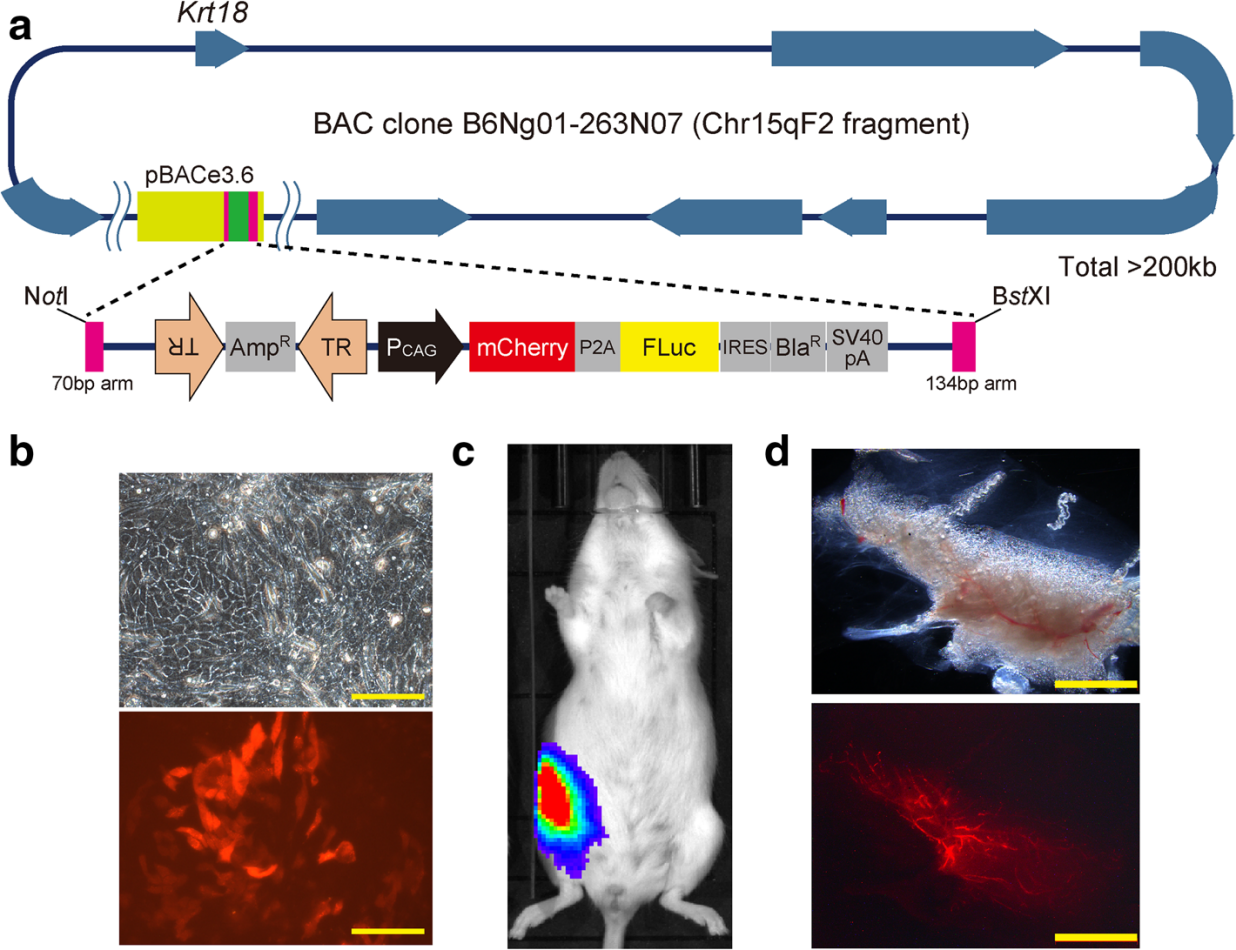
High loading capacity of the *piggyBac* donor vector for reconstitution of vector-introduced mammary glands. **a** Map of *piggyBac* transposon donor vector carrying long a DNA (approximately 200 kb) derived from a bacterial artificial chromosome (BAC) vector (RIKEN B6Ng01-263 N07) containing a fragmentary mouse genomic sequence encompassing at least eight known genes including *Krt18*. **b** Microscopic view of the BAC vector-introduced MECs. Red fluorescence (lower panels) shows mCherry marker driven by CAG promoter. Scale bar = 100 μm. **c** A representative image showing luciferase activity of mammary outgrowths at day 50 after mammary epithelial cell injection into cleared fat pad. **d** Microscopic view of resected fat pads from **c**. Red fluorescence (lower panels) shows mCherry marker expression. Scale bar = 5 mm

Viral vector systems have a length restriction for carrying or packaging a gene (maximum of ~ 10 kb), whereas transposon vectors have a much higher capacity [25–27]. We investigated whether the length restriction of viral vector systems can be overcome by our *piggyBac* transposon vector system. We constructed a donor vector that can load DNA elements from a BAC clone (B6Ng01-263 N07) obtained from RIKEN BRC, whose vector size was > 200 kb (Fig. 5a). When we introduced this vector into an MaSC-enriched fraction using electroporation, cultured MECs expressed mCherry fluorescence (Fig. 5b). In addition, outgrowth of BAC-introduced mammary glands was detected in vivo and ex vivo based on Fluc and mCherry marker gene expression, respectively (Fig. 5c, d). This suggested that our vector system can deliver much larger cargo into cells compared with conventional viral vector systems.

### *PyMT* oncogene expression in mammary glands phenocopied the MMTV-*PyMT* transgenic mouse model

**Fig. 6:**
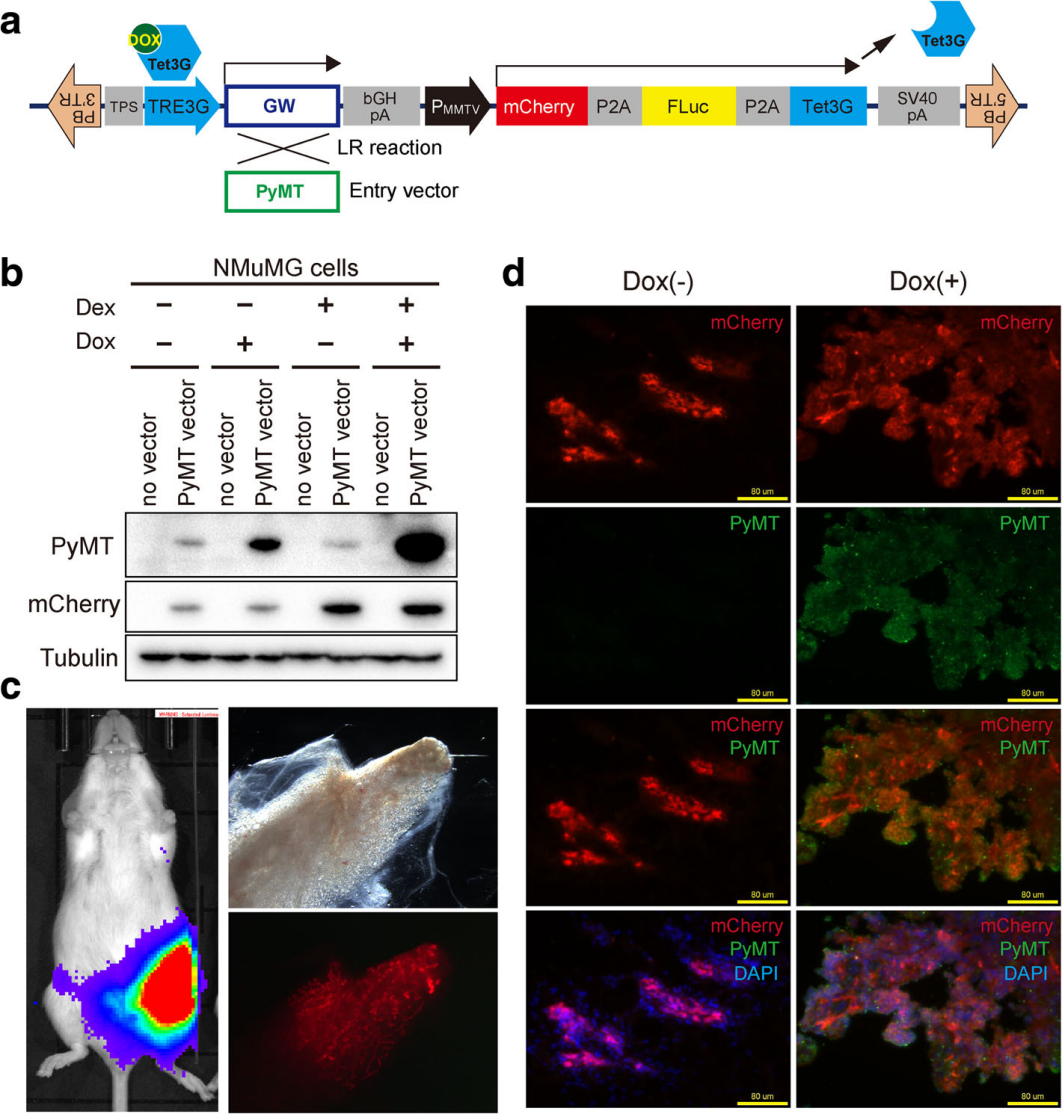
Establishment of *PyMT* oncogene-introduced mammary glands. **a** Vector construct of *piggyBac* transposon donor vector expressing mCherry, firefly luciferase (FLuc), and Tet3G under the control of the mouse mammary tumor virus (MMTV) promoter. Polyoma-virus middle T antigen (*PyMT*) was introduced downstream of TRE3G using LR recombinase of the Gateway system. **b** Immunoblot analysis of indicated lysates derived from NMuMG cells not transfected or transfected with TRE-PyMT vector. Tubulin represents internal control. **c** (left panel) A representative image showing luciferase activity of mammary outgrowths at day 42 after mammary epithelial cell injection into cleared fat pad. (Right panels) Microscopic view of resected fat pads. Red fluorescence (lower panel) shows mCherry marker expression. Scale bar = 5 mm. **d** Analysis of PyMT expression by immunofluorescence staining. Frozen sections from TRE-PyMT-introduced mammary glands passaged from a primarily reconstituted gland without Dox (right panels) and with Dox (left panels) in drinking water were co-immunostained with antibodies against mCherry (red, upper panels) and PyMT (green). Scale bars = 80 μm. DAPI 4′,6-diamidino-2-phenylindole, Dex dexamethasone, Dox doxycycline

We next investigated whether our vector system could phenocopy a transgenic mouse model of breast cancer. It is known that mice that express *PyMT* under control of the MMTV promoter develop hyperplasias, which progress to adenocarcinoma [35, 36]. We designed the *piggyBac* transposon vector, which can express *PyMT* by the addition of Dox using Tet3G of Tet-On system that exhibits expression under the control of the MMTV promoter (Fig. 6a).

To check whether the inducible expression vector for *PyMT* functions properly, we transfected it into NMuMG cells and analyzed the protein expression by immunoblotting. To activate MMTV promoter in NMuMG cells, a synthetic glucocorticoid, dexamethasone (Dex), was added into the culture medium [37]. As expected, Dex- and Dox-dependent PyMT upregulation was observed (Fig. 6b). It was also observed that PyMT was weakly expressed without the addition of Dox (lanes 2 and 6).

**Fig. 7:**
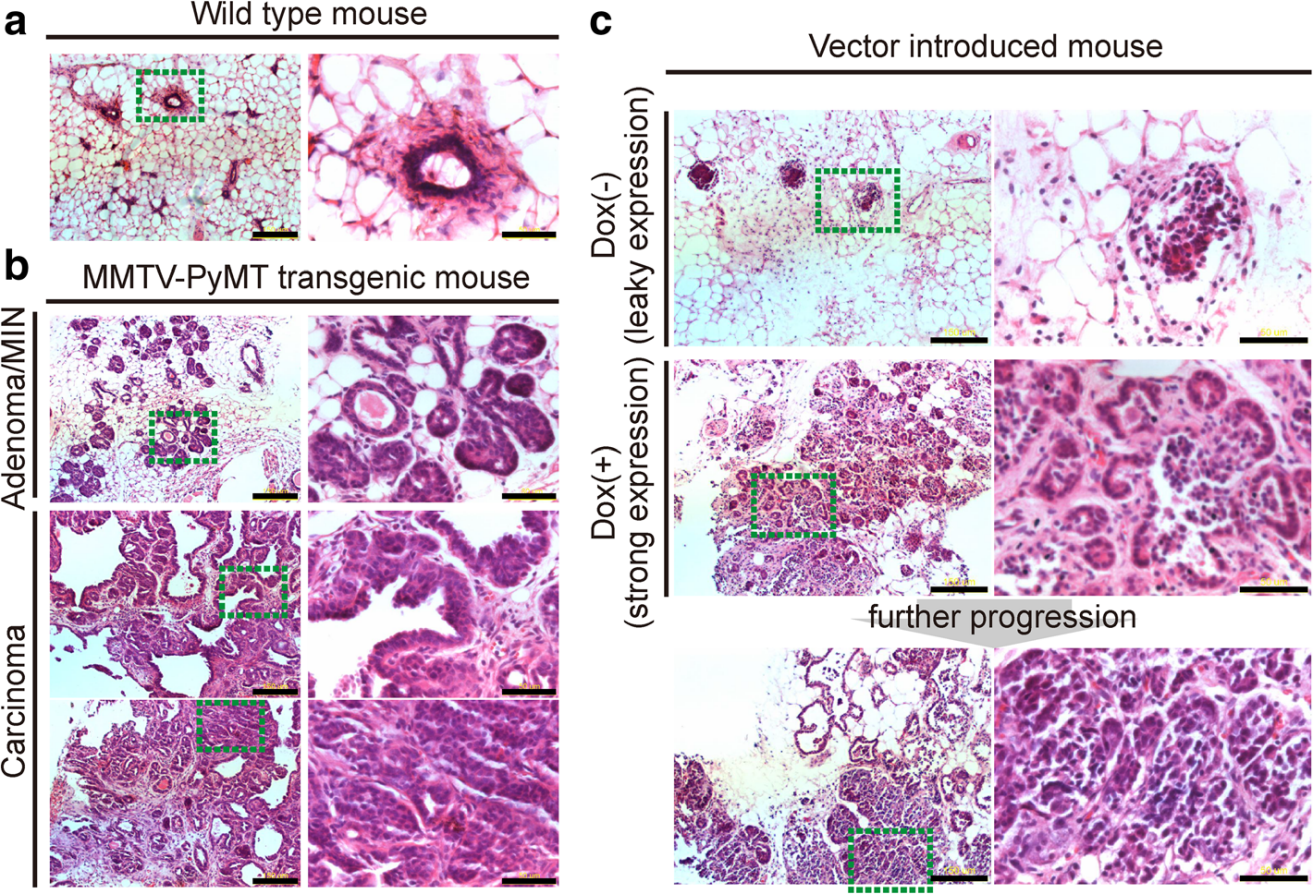
Analysis of the histochemical properties of the oncogene-induced mammary glands. Images of hematoxylin and eosin staining of paraffin sections of **a** normal and **b** mouse mammary tumor virus-polyoma-virus middle T antigen (MMTV-*PyMT*) transgenic mouse mammary glands. Scale bars = 150 μm (left panels) and 50 μm (right panels). The right panels represent a higher resolution image corresponding to the green dotted square in the left panels. **c** Representative images of hematoxylin and eosin staining of paraffin sections of TRE-*PyMT*-introduced mammary glands fed without doxycycline (Dox) (upper panels) or with Dox (middle panels). The right panels represent a higher resolution image corresponding to the green dotted square in the left panels. The lower panels show one of the progressed tumors derived from the same tissue as shown in the upper panels. Scale bars = 150 μm (left panels) and 50 μm (right panels)

We transduced PyMT vector into MaSC-enriched MECs (Fig. 6c) and passaged its reconstituted tissues about 2 months after primary transplantation. The secondary transplanted mice were fed with drinking water with or without doxycycline 2–3 weeks after transplantation for about 3 months. In histochemical analyses of the dissected tissues, we detected the co-expression of PyMT with mCherry (Fig. 6d) and morphological changes that were highly similar to those of tissues from MMTV-PyMT transgenic mice (Fig. 7a-c). The adenoma and carcinoma-like phenotype depended on PyMT expression levels observed in Fig. 6b; namely, a weak phenotype was observed on leaky-level expression (Dox(−)) or a strong phenotype on high expression (Dox(+)). These results demonstrated that the novel method that we developed provides a promising alternative to producing transgenic mice.

## Discussion

The mouse mammary gland provides a unique model for the study of MaSCs and differentiation pathways and pathogenesis of breast cancer [38–40]. Recent studies of cellular hierarchical issues using mammary glands have contributed to better understanding of “cells of origin” and “cancer stem cells” with regard to breast cancer [5, 40–43]. Breast cancer occurs when genetic mutations occur during tissue development. Transgenic and knockout mouse models have been used in previous studies involving genetic analysis. By several in vitro, allograft, and xenograft screenings and analyses of microarray data, we found multiple candidate genes and hoped to evaluate their oncogenic or malignant phenotypes in vivo on mammary epithelium at specific differentiation phases [6–9]. For practicality of this situation, an alternative method needs to be developed for producing genetically manipulated mammary glands with ease and rapidity. Combined with *piggyBac* transposon vector system and electroporation, we obtained genetically manipulated mammary gland in 2–3 months. Although, the reconstitution efficiency of the first genetically manipulated mammary gland was low (3/18 to 7/18) (Table 1), secondary transplantation after cutting the first reconstituted mammary glands into small pieces and selecting gene-introduced mammary gland using the mCherry fluorescence marker yielded successful expansion with high efficiency (11/16 to 12/14 from one regenerated mammary gland). This property is highly useful for statistical analyses or maintenance of mammary tissue lines. Doxycycline-dependent change in expression levels was successfully achieved in a case where an EGFP- or PyMT-inducible expression cassette was used in the vector. In future studies, we hope to apply this tool to candidate genes that may confer malignant phenotypes and evaluate their in-vivo function during the development of mammary gland. By using IVIS, events such as metastasis can be monitored by our vector system. In addition, the expression promoter presented here can be modified depending on the purpose of the particular research. For example, by utilizing the K8 or K14 promoter, lineage-specific expression control in luminal or basal cells, respectively, can be achieved. In addition, it is also possible to establish a gene suppression system by introducing CRISPR-Cas9 or a knockdown approach. These modifications are easily achieved thanks to the use of the transposon donor vector backbone, which has almost no limitation concerning DNA complexity (termination or promoter sequence) and length. Actually, our system enabled us to produce a mammary gland into which a BAC vector was integrated whose vector size was > 200 kb (Fig. 5), suggesting the possibility of using genomic DNA elements of almost unlimited length for gene transduction. Thus, the presented methods and tools may be broadly applicable and open a new avenue for breast cancer research.

## Conclusions

With our system presented here, gene transduction into mammary gland in vivo can be easily and quickly achieved and gene expression can be controlled by administering doxycycline. This system for genetic manipulation is potentially useful for analyzing genes involved in breast cancer.

## Additional file

Additional file 1: **Table S1.** Electroporation parameters obtained by NEPA21 electroporator (NEPAGENE) for gene transduction into MaSC-enriched cells. **Figure S1.** Isolation of basal/MaSC fraction from female mice aged 8–10 weeks. **a** Singlet sorting. **b** Further singlet sorting. **c** Lin(−) 7-AAD(−) sorting, excluding hematopoietic, endothelial, and stromal cells (Lin(+)), and dead cells (7-AAD(+)). **d** Sorting of basal/MaSC fraction by CD49f and CD24. **Figure S2.** Dox-dependent expression of TRE3G-EGFP gene-introduced MECs under MMF culture. Red fluorescence shows mCherry marker driven by P_CAG_. Scale bar = 500 μm. (DOCX 1884 kb)

## Abbreviations

7-AAD: 7-Amino-actinomycin D
BAC: Bacterial artificial chromosome
CAG: Cytomegalovirus early enhancer/chicken β-actin
DAPI: 4′,6-Diamidino-2-phenylindole
Dex: Dexamethasone
DMEM: Dulbecco’s modified Eagle’s medium
Dox: Doxycycline
EGFP: Enhanced green fluorescent protein
FACS: Fluorescence-activated cell sorting
FBS: Fetal bovine serum
Fluc: Firefly luciferase
ITR: Inverted terminal repeat
IVIS: In-vivo imaging system
MaSC: Mammary stem cell
MEC: Mammary epithelial cell
MFG: Milk fat globule
MMTV: Mouse mammary tumor virus
PBS: Phosphate-buffered saline
PyMT: Polyoma-virus middle T antigen
ROCKi: Rho-associated coiled-coil-forming kinase inhibitor
