# Supplementary material for: A method of producing genetically manipulated mouse mammary gland
Source: Breast Cancer Res. 2019 Jan 5;21:1. doi: 10.1186/s13058-018-1086-8 (PMC6321679; doi:10.1186/s13058-018-1086-8)
Supplement: Supplementary file 1 — Table S1. Electroporation parameters obtained by NEPA21 electroporator (NEPAGENE) for gene transduction into MaSC-enriched cells. Figure S1. Isolation of basal/MaSC fraction from female mice aged 8–10 weeks. a Singlet sorting. b Further singlet sorting. c Lin(−) 7-AAD(−) sorting, excluding hematopoietic, endothelial, and stromal cells (Lin(+)), and dead cells (7-AAD(+)). d Sorting of basal/MaSC fraction by CD49f and CD24. Figure S2. Dox-dependent expression of TRE3G-EGFP gene-introduced MECs under MMF culture. Red fluorescence shows mCherry marker driven by PCAG. Scale bar = 500 μm. (DOCX 1884 kb) [file 13058_2018_1086_MOESM1_ESM.docx]

**Supplementary Figure**

**Table S1** Electroporation parameters by NEPA21 electroporator (NEPAGENE) for transgene into MaSC-enriched cells

| Pulse voltage  (V) | Pulse width  (ms) | Pulse intervals  (ms) | Pulse  numbers  (times) | Pulse  decay  (%) | Polarity |
| --- | --- | --- | --- | --- | --- |
| Poring pulse | | | | | |
| 200 | 2.5 | 50 | 2 | 10 | + |
| Transfer pulse | | | | | |
| 20 | 50 | 50 | 5 | 40 | +/- |


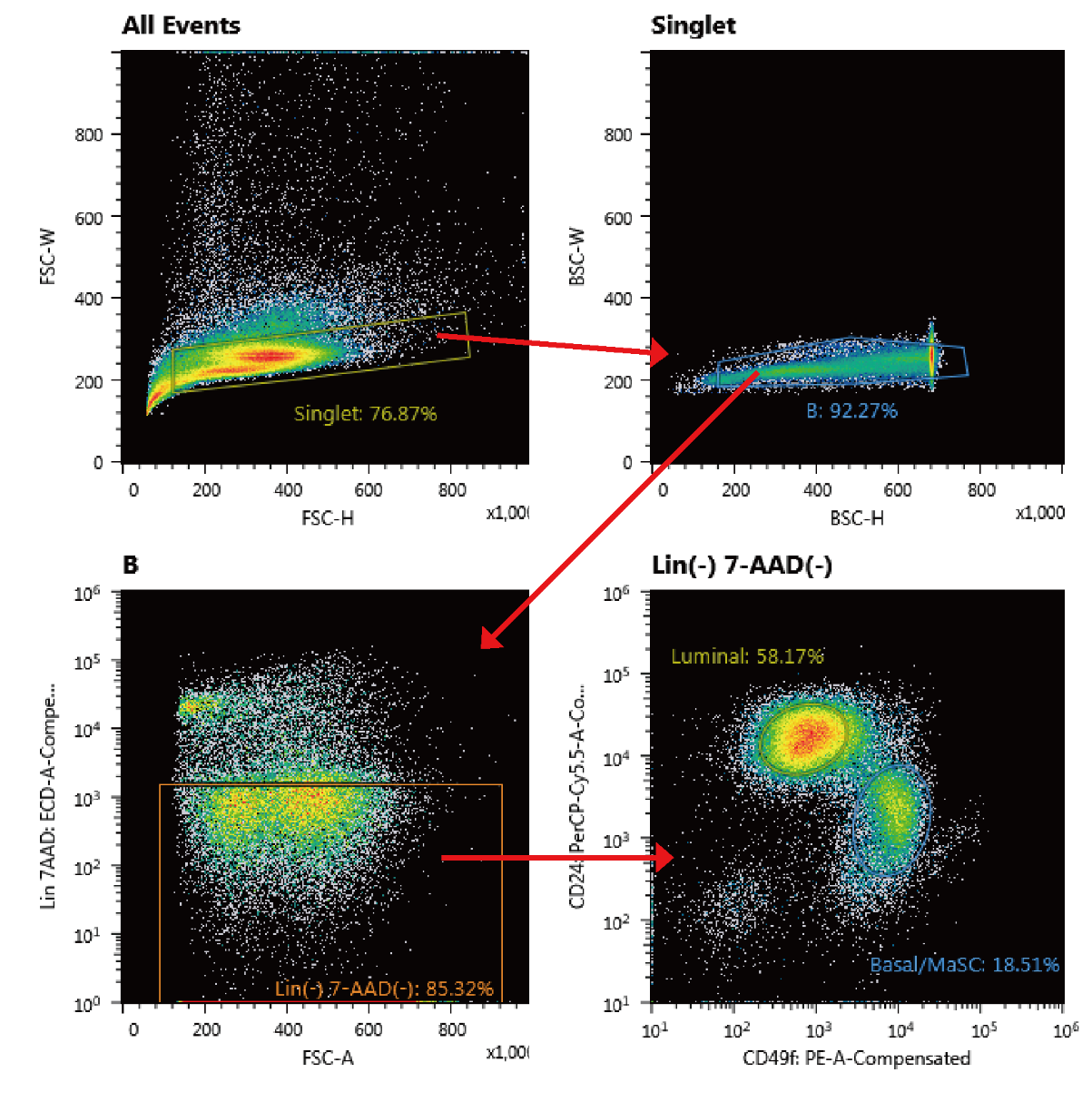


**Figure S1** Isolation of basal/MaSC fraction from female mice aged from 8 to 10 weeks. **a.** Singlet sorting. **b.** Further singlet sorting. **c.** Lin(−) 7-AAD(−) sorting, excluding hematopoietic, endothelial, and stromal cells [Lin(+)], and dead cells [7-AAD(+)]. d. Sorting of basal/MaSC fraction by CD49f and CD24.


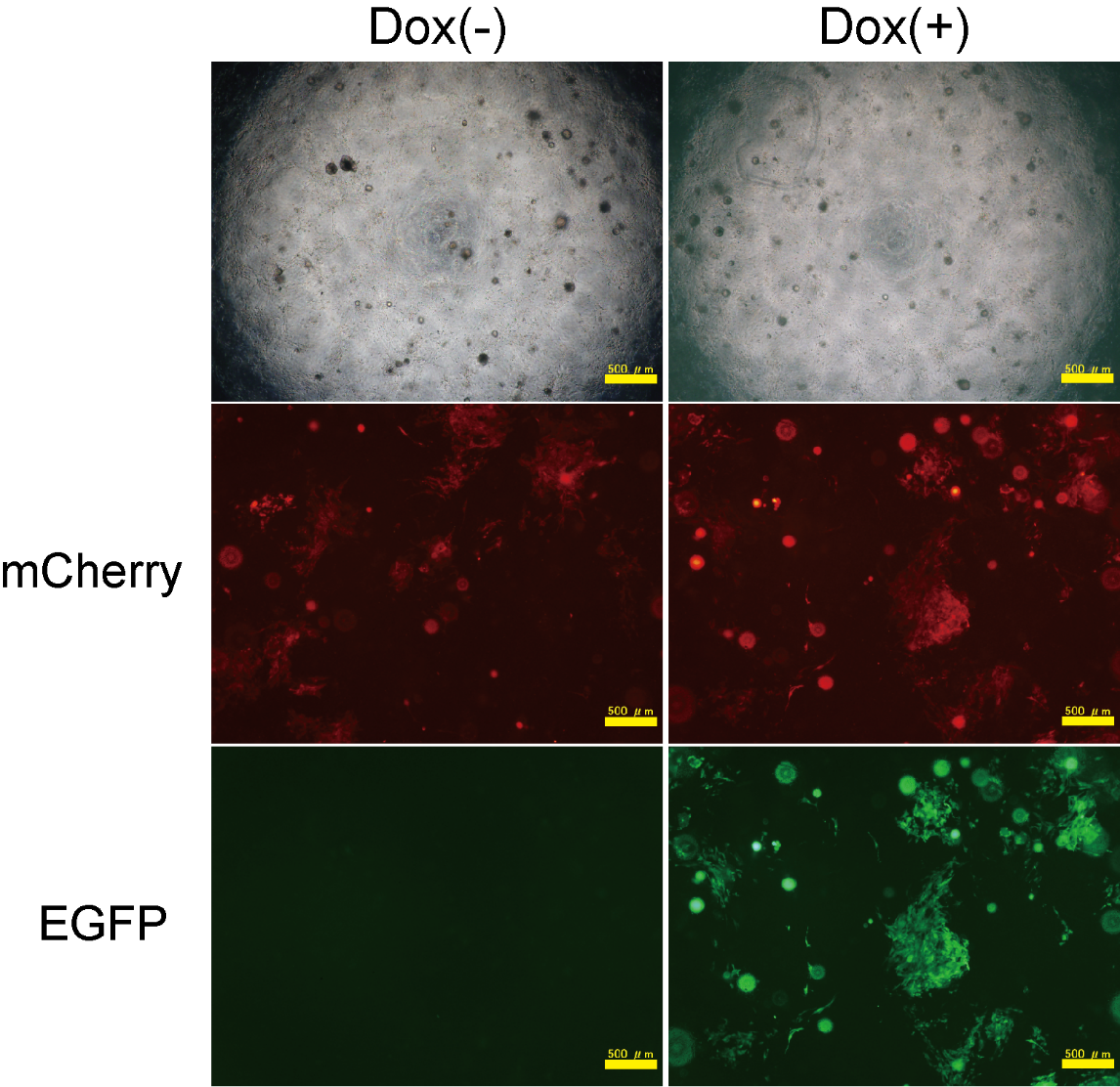


**Figure S2** Dox-dependent expression of TRE3G-EGFP gene-introduced MECs under MMF culture. Red fluorescence shows mCherry marker driven by P_CAG_. Scale bar = 500 µm.
